# The influence of ovarian cyst type and size on ovarian reserve markers: implications for fertility counseling and preservation strategy

**DOI:** 10.3389/fendo.2025.1517789

**Published:** 2025-06-06

**Authors:** Xingyu Sun, Doudou Liu, Zijing Guo, Lijuan He, Shaohua Wang

**Affiliations:** ^1^ Department of Gynecology, The Affiliated Traditional Chinese Medicine Hospital, Southwest Medical University, Luzhou, Sichuan, China; ^2^ Reproductive Medicine Center, The Affiliated Hospital, Southwest Medical University, Luzhou, Sichuan, China; ^3^ School of Clinical Medicine, North Sichuan Medical College, Nanchong, Sichuan, China; ^4^ Department of Health Management Center, The Affiliated Hospital, Southwest, Medical University, Luzhou, Sichuan, China; ^5^ Department of Pathology, The Affiliated Hospital, Southwest Medical University, Luzhou, China

**Keywords:** ovarian cysts, ovarian reserve, AMH, FSH, AFC, endometrioma, fertility preservation

## Abstract

**Background:**

Ovarian cysts are common in reproductive-aged women and may affect ovarian reserve, thereby impacting fertility potential. This study aimed to evaluate the differential impact of various ovarian cyst types and sizes on ovarian reserve markers to inform individualized fertility care.

**Methods:**

A retrospective cohort study was conducted on 474 women classified into four groups: endometrioma, simple cyst, dermoid cyst, and controls without cysts. Serum anti-Müllerian hormone (AMH), follicle-stimulating hormone (FSH) levels, and antral follicle count (AFC) were assessed. Multivariate regression analyses adjusted for age and BMI were used to evaluate the independent effects of cyst type and size on ovarian reserve markers.

**Results:**

Compared to controls, women with ovarian cysts had significantly lower AMH and AFC levels and higher FSH levels (all p < 0.001). Endometriomas exerted the most profound negative impact, with the lowest AMH and AFC values and the highest FSH levels. Larger cysts were independently associated with greater reductions in AMH and AFC and increases in FSH levels.

**Conclusion:**

Both ovarian cyst type and size significantly influence ovarian reserve. Endometriomas and larger cysts are particularly detrimental. These findings highlight the need for personalized fertility counseling and early preservation strategies in affected women.

## Introduction

Ovarian cysts are a frequent gynecological condition, particularly among women of reproductive age. These fluid-filled sacs, either within or on the surface of the ovary, vary in type, with common forms including endometriomas, simple cysts, and dermoid cysts. While many ovarian cysts are asymptomatic and benign, certain types, especially endometriomas, have been associated with adverse effects on reproductive health, including fertility impairment (1, 2).

Ovarian reserve, a critical factor in female fertility, reflects the ovary’s capacity to produce viable oocytes for fertilization. Key markers used to assess ovarian reserve include Anti-Müllerian Hormone (AMH), Follicle-Stimulating Hormone (FSH), and Antral Follicle Count (AFC) (3, 4). A decline in these markers can indicate reduced ovarian reserve, which is closely linked to fertility potential (5, 6). The relationship between ovarian cysts and ovarian reserve has gained increasing attention in recent years, as both cyst type and size may influence the function of the ovarian follicle pool (7).

While studies have demonstrated that ovarian cysts, particularly endometriomas, can negatively affect AMH and AFC, the specific impact of cyst type and size on ovarian reserve markers remains under debate (8, 9). Ovarian endometriomas are reported to affect approximately 17–44% of women with endometriosis (10), and may reduce AMH levels by up to 30% compared to healthy controls (11). This reduction is likely related to cortical fibrosis, follicular atresia, and local inflammation within the affected ovary. Although previous studies have suggested that larger cysts or those associated with endometriosis may exert a greater detrimental impact on ovarian function (12, 13), there is a paucity of studies directly comparing the effects of different cyst types and sizes on ovarian reserve under standardized clinical and methodological conditions. This research gap limits clinicians’ ability to make evidence-based decisions on individualized fertility counseling and intervention.

This study aims to fill this gap by systematically evaluating the relationship between ovarian cyst type, cyst size, and ovarian reserve indicators. By analyzing a cohort of patients with endometriomas, simple cysts, dermoid cysts, and a control group, we seek to clarify the extent to which these cysts impact AMH, FSH, and AFC levels. This research is crucial for informing fertility management and optimizing treatment strategies for women with ovarian cysts, particularly those planning for pregnancy or undergoing fertility treatments.

## Materials and methods

### Study design

This was a retrospective cohort study designed to evaluate the relationship between ovarian cyst type and size and ovarian reserve markers in women of reproductive age. The study was conducted at the The Affiliated Hospital, Southwest Medical University, using patient records from January 2020 to December 2022. As this was a retrospective cohort study, no *a priori* sample size calculation was performed. Instead, all eligible patients meeting the inclusion criteria during the defined recruitment period (January 2020 to December 2022) were included. The final sample size (n = 474) was sufficient to achieve statistical significance in the primary analyses, as evidenced by the reported p-values. This study was reported in accordance with the STROBE (Strengthening the Reporting of Observational Studies in Epidemiology) statement, following the guidance of the EQUATOR (Enhancing the QUAlity and Transparency Of health Research) Network. A completed STROBE checklist is provided as a **Supplementary File**.

### Participants

A total of 474 women aged 18 to 50 years were included in the study. All participants were recruited during routine outpatient gynecological consultations at The Affiliated Hospital, Southwest Medical University, where they underwent pelvic ultrasound and hormonal evaluation as part of their clinical assessment. Participants were categorized into four groups based on their cyst type: endometrioma (n = 65), simple cyst (n = 132), dermoid cyst (n = 43), and a control group without ovarian cysts (n = 234). Inclusion criteria were: women aged 18 to 50 years; a confirmed diagnosis of ovarian cyst via transvaginal ultrasound; no prior history of ovarian surgery; no hormonal therapy (e.g., oral contraceptives or ovulation induction agents) within six months prior to evaluation; and no known genetic, metabolic, or endocrine disorders (such as Turner syndrome, PCOS, or thyroid dysfunction) that could affect reproductive function. Exclusion criteria included: a history of ovarian or pelvic surgery; use of hormonal medications within six months of enrollment; presence of systemic diseases or medical conditions known to impact ovarian reserve (e.g., chemotherapy exposure or autoimmune disorders); and incomplete clinical records or missing laboratory/imaging data. Simple ovarian cysts were defined as unilocular, anechoic, thin-walled structures with smooth internal walls and no internal septations, solid components, or papillary projections, as observed on transvaginal ultrasound. These features are consistent with benign functional cysts such as follicular cysts or corpus luteum cysts. The control group consisted of age-matched women without ovarian cysts. Age matching was conducted using frequency matching in 5-year age intervals to ensure that the age distribution of the control group was comparable to that of the cyst groups. This approach allowed for group-level comparability while maintaining statistical power. No individual pair matching or propensity score matching was performed.

### Data collection

Clinical and demographic data were extracted from electronic medical records. This included patient age, body mass index (BMI), menstrual cycle characteristics, obstetric history, presence of chronic diseases, smoking and alcohol use, and family history of reproductive issues. Ovarian cysts were classified as endometrioma, simple cyst, or dermoid cyst primarily based on transvaginal ultrasound findings. Diagnosis was made according to standardized sonographic criteria. Endometriomas were identified by the presence of homogeneous, low-level internal echoes (“ground-glass” appearance) within unilocular cysts. Simple cysts were diagnosed as unilocular, anechoic, thin-walled cysts without septations or solid components. Dermoid cysts (mature cystic teratomas) were diagnosed by the presence of echogenic nodules (Rokitansky nodule), shadowing, or heterogeneous components suggestive of fat or calcification. In a minority of cases where surgical removal was performed, histopathological confirmation was available and consistent with ultrasound findings.

### Ovarian reserve assessment

Ovarian reserve markers were assessed by measuring serum levels of Anti-Müllerian Hormone (AMH), Follicle-Stimulating Hormone (FSH), and Antral Follicle Count (AFC). Blood samples for AMH and FSH were collected in the early follicular phase (days 2–4 of the menstrual cycle) as part of the routine clinical evaluation for ovarian reserve conducted during outpatient gynecological visits at The Affiliated Hospital, Southwest Medical University. These hormonal assessments are standard practice for patients presenting with ovarian cysts or undergoing fertility assessment.

### Study outcomes

The primary outcomes of this study were the levels of ovarian reserve markers, including serum Anti-Müllerian Hormone (AMH), Follicle-Stimulating Hormone (FSH), and Antral Follicle Count (AFC), in relation to different types of ovarian cysts (endometrioma, simple cyst, dermoid cyst) and in comparison with a control group. Secondary outcomes included the correlation between cyst size and ovarian reserve markers, and the evaluation of whether cyst size independently predicted alterations in AMH, FSH, and AFC levels after adjusting for age and BMI.

### Statistical analysis

Descriptive statistics were used to summarize the clinical and demographic characteristics of the study groups. The normality of continuous variables was tested using the Shapiro-Wilk test. Mean and standard deviations were reported for normally distributed variables, while medians and interquartile ranges were used for non-normally distributed variables. Comparisons between groups were made using ANOVA or Kruskal-Wallis tests for continuous variables, and chi-square tests for categorical variables.

Pearson’s correlation coefficients (R) were calculated to assess the relationships between cyst size and ovarian reserve markers (AMH, FSH, and AFC). Multiple linear regression models were employed to evaluate the independent effects of cyst size on these markers, adjusting for potential confounding variables such as age and BMI. A p-value of <0.05 was considered statistically significant. All statistical analyses were performed using SPSS software (version 25.0, IBM Corp).

## Results

### Clinical and demographic characteristics

**Figure 1:**
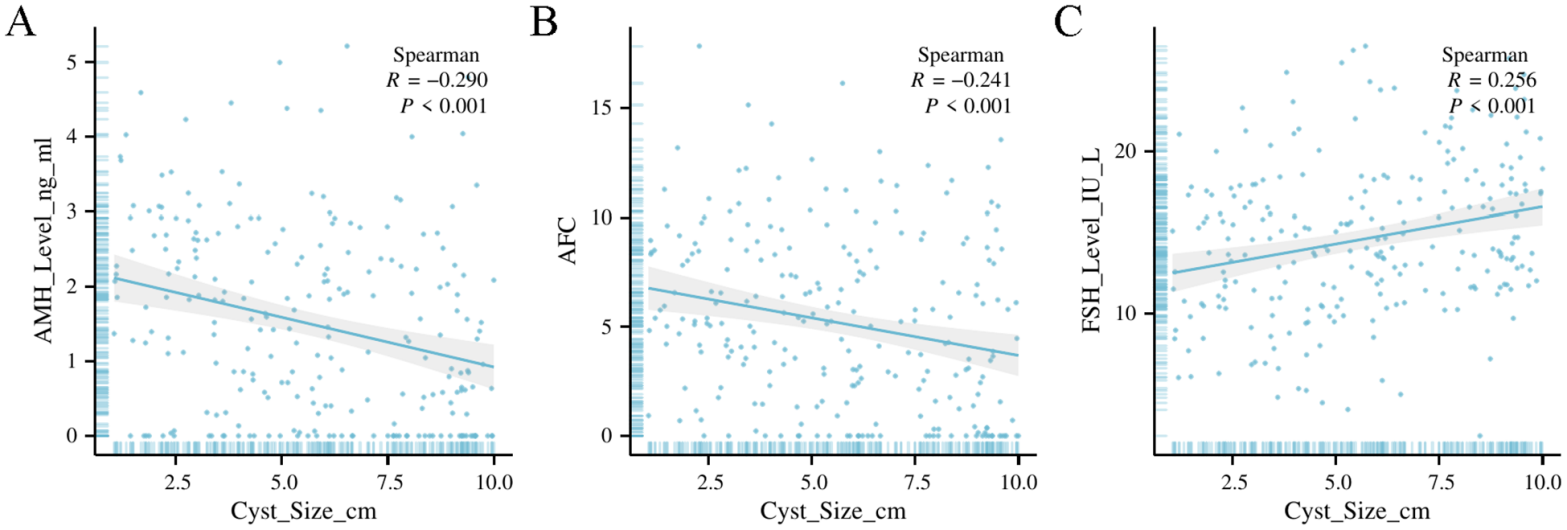
Clinical and demographic characteristics of patients with different types of ovarian cysts. **(A)** Age distribution across the different groups (endometrioma, simple cyst, dermoid cyst, and control group) showing median age and interquartile range (IQR). No significant difference was observed between groups (p=0.966p = 0.966p=0.966). **(B)** Body Mass Index (BMI) distribution by cyst type, illustrating similar BMI across all groups with no statistically significant difference (p=0.494p = 0.494p=0.494). **(C)** Menstrual cycle length in days for each cyst type, showing comparable cycle lengths among groups with no significant variation (p=0.478p = 0.478p=0.478).

A total of 474 women were included in the study: 65 with endometriomas, 132 with simple cysts, 43 with dermoid cysts, and 234 in the control group. The median age across all groups ranged from 33 to 35 years, with no statistically significant differences observed (p = 0.268). Similarly, no significant differences were found in body mass index (BMI), menstrual cycle length, number of pregnancies, or history of miscarriage (all p > 0.05), as summarized in **Table 1** and illustrated in **Figures 1A–C**.

**Table 1:** Clinical and demographic characteristics of patients with different types of ovarian cysts.

Characteristics	Endometrioma	Simple Cyst	Dermoid Cyst	None	P value
n	65	132	43	234	
Age, median (IQR)	35 (27, 41)	34.5 (24, 42)	33 (23.5, 41.5)	34 (27, 43)	0.966
Cyst_Size_cm, mean ± sd	5.7695 ± 2.7802	5.7114 ± 2.5485	5.442 ± 2.7318	0 ± 0	NaN
AMH_Level_ng_ml, median (IQR)	1.1701 (0, 2.334)	1.3399 (0.42956, 2.4112)	1.8279 (0.23442, 2.5722)	4.6093 (3.4629, 5.5235)	< 0.001
FSH_Level_IU_L, mean ± sd	14.081 ± 4.1336	15.226 ± 4.9872	13.569 ± 4.6861	6.8879 ± 2.8608	< 0.001
AFC, median (IQR)	5.4441 (1.473, 9.0037)	4.7566 (1.587, 7.793)	5.2602 (1.9851, 7.7097)	14.944 (11.71, 19.068)	< 0.001
BMI, median (IQR)	25.767 (21.763, 30.356)	26.845 (21.967, 31.113)	27.211 (22.282, 31.143)	26.751 (22.637, 30.985)	0.494
Menstrual_Cycle_Length_days, median (IQR)	29 (26, 32)	29 (25, 32)	27 (25, 32)	28 (24, 31)	0.478
Days_Of_Menstruation, median (IQR)	5 (4, 7)	5 (3.75, 6)	5 (4, 6)	5 (3, 6)	0.300
Pregnancies, median (IQR)	2 (1, 3)	2 (1, 3)	3 (1, 3.5)	2 (1, 3)	0.715
Miscarriages, n (%)					0.455
1	24 (5.1%)	44 (9.3%)	10 (2.1%)	93 (19.6%)	
2	21 (4.4%)	41 (8.6%)	15 (3.2%)	61 (12.9%)	
0	20 (4.2%)	47 (9.9%)	18 (3.8%)	80 (16.9%)	
Family_History_Reproductive_Issues, n (%)					0.145
No	54 (11.4%)	95 (20%)	35 (7.4%)	190 (40.1%)	
Yes	11 (2.3%)	37 (7.8%)	8 (1.7%)	44 (9.3%)	
Estrogen_Level_pg_ml, median (IQR)	237.66 (138.96, 316.8)	214.94 (124.11, 290.55)	200.14 (79.007, 300.76)	190.89 (102.05, 275.25)	0.366
Progesterone_Level_ng_ml, median (IQR)	23.73 (11.36, 31.677)	19.648 (9.3607, 27.947)	21.237 (7.4923, 30.274)	19.082 (9.9674, 30.221)	0.428
Surgical_History, n (%)					0.059
Yes	28 (5.9%)	32 (6.8%)	12 (2.5%)	72 (15.2%)	
No	37 (7.8%)	100 (21.1%)	31 (6.5%)	162 (34.2%)	
Smoking, n (%)					0.069
No	58 (12.2%)	107 (22.6%)	36 (7.6%)	176 (37.1%)	
Yes	7 (1.5%)	25 (5.3%)	7 (1.5%)	58 (12.2%)	
Alcohol_Consumption, n (%)					0.086
No	42 (8.9%)	61 (12.9%)	23 (4.9%)	114 (24.1%)	
Yes	23 (4.9%)	71 (15%)	20 (4.2%)	120 (25.3%)	
Chronic_Diseases, n (%)					0.098
No	50 (10.5%)	102 (21.5%)	35 (7.4%)	159 (33.5%)	
Yes	15 (3.2%)	30 (6.3%)	8 (1.7%)	75 (15.8%)	

n, Number of patients; IQR, Interquartile Range; sd, Standard Deviation; AMH, Anti-Müllerian Hormone; FSH, Follicle-Stimulating Hormone; AFC, Antral Follicle Count; BMI, Body Mass Index; P value, Statistical significance (probability value); Endometrioma, Simple Cyst, Dermoid Cyst, None, Types of ovarian cysts; Estrogen_Level_pg_ml, Estrogen Level in pg/ml; Progesterone_Level_ng_ml, Progesterone Level in ng/ml; Surgical History, Smoking, Alcohol Consumption, Chronic Diseases, Categorical variables (Yes/No).

### Ovarian reserve markers by cyst type

**Figure 2:**
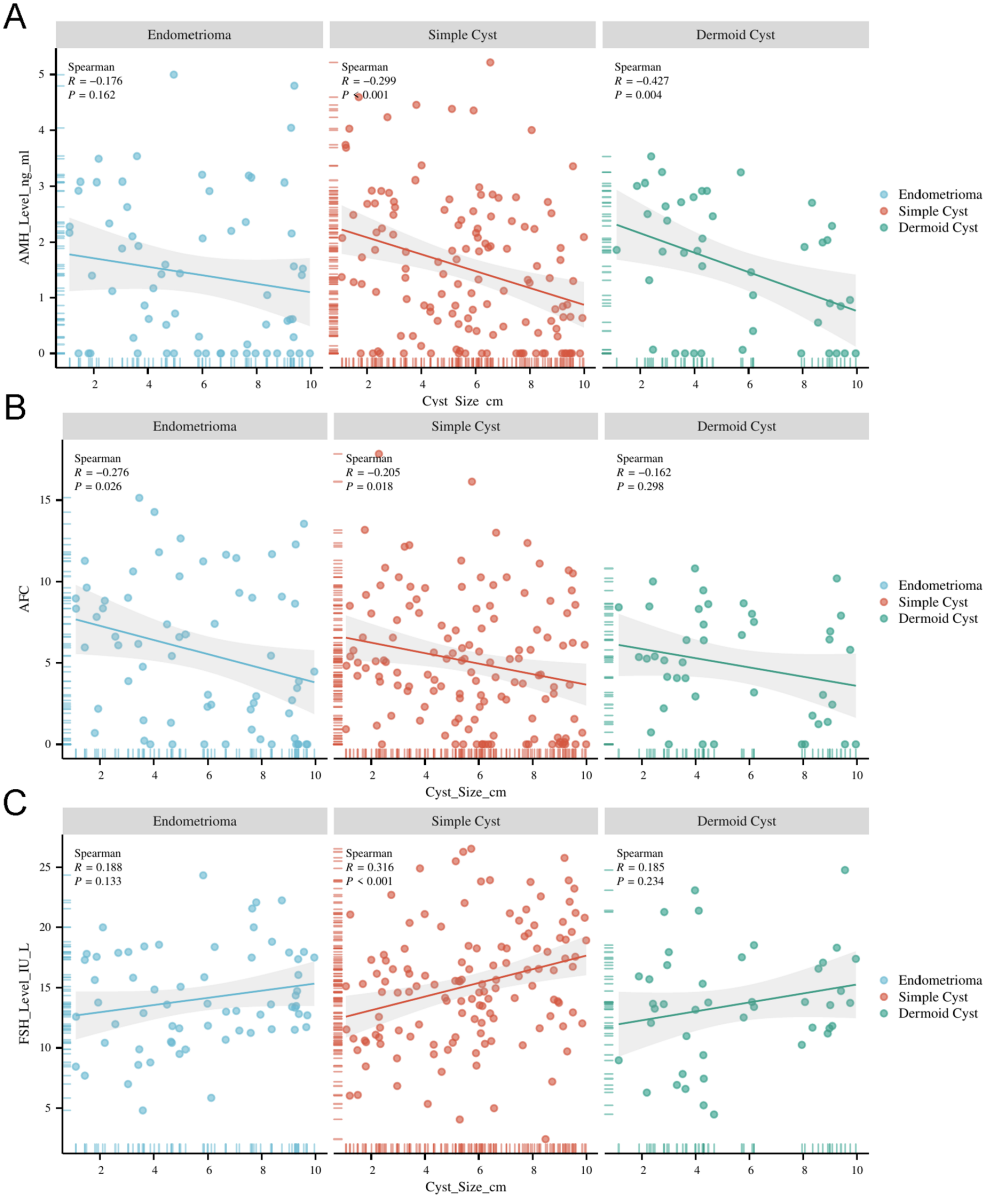
Ovarian reserve markers by cyst type. **(A)** Median Anti-Müllerian Hormone (AMH) levels across different cyst types, showing significantly lower levels in patients with endometriomas, simple cysts, and dermoid cysts compared to the control group (p<0.001p < 0.001p<0.001). **(B)** Mean Follicle-Stimulating Hormone (FSH) levels by cyst type, indicating significantly higher FSH levels in patients with ovarian cysts, especially in those with endometriomas (p<0.001p < 0.001p<0.001). **(C)** Median Antral Follicle Count (AFC) for each cyst group, demonstrating reduced AFC in cyst groups, particularly in endometriomas, compared to controls (p<0.001p < 0.001p<0.001).

Significant differences in ovarian reserve markers were observed between the cyst groups and the control group (**Table 1**, **Figure 2**). The median AMH levels were lowest in the endometrioma group (1.17 ng/mL), followed by the simple cyst (1.34 ng/mL) and dermoid cyst (1.83 ng/mL) groups, all significantly lower than the control group (4.61 ng/mL) (p < 0.001). FSH levels were significantly elevated in the cyst groups, with endometriomas showing the highest mean FSH (14.08 IU/L), followed by simple cysts (15.23 IU/L) and dermoid cysts (13.57 IU/L), compared to controls (6.89 IU/L) (p < 0.001). AFC values showed a corresponding decrease, with the lowest median count in the endometrioma group (5.44), followed by simple cysts (4.76) and dermoid cysts (5.26), versus the control group (14.94) (p < 0.001). To validate these findings, a second measurement set confirmed the same trends: AMH remained lowest in the endometrioma group (1.32 ng/mL), followed by simple cysts (1.88 ng/mL) and dermoid cysts (2.13 ng/mL), compared to controls (3.05 ng/mL). FSH was again highest in the endometrioma group (10.4 mIU/mL), followed by simple (8.7 mIU/mL) and dermoid cysts (8.1 mIU/mL), and lowest in controls (6.5 mIU/mL). AFC remained significantly reduced in all cyst groups compared to controls (p < 0.001).

### Correlation between cyst size and ovarian reserve markers

Cyst size was significantly correlated with ovarian reserve markers (**Table 2**). AMH and AFC were negatively correlated with cyst size (R = –0.290 and –0.241, respectively; both p < 0.001), indicating that larger cysts were associated with diminished ovarian reserve. Conversely, FSH was positively correlated with cyst size (R = 0.256, p < 0.001). These correlations were most pronounced in the endometrioma subgroup.

**Table 2:** Correlation analysis between ovarian cyst characteristics and ovarian reserve markers.

Variables	AMH		FSH		AFC	
	*R*	*P*	*R*	*P*	*R*	*P*
Cyst_Size	-0.290	<0.001	0.256	<0.001	-0.241	<0.001
Endometrioma- Cyst_Size	-0.176	0.162	0.188	0.133	-0.276	0.026
Endometrioma-Simple Cyst	-0.299	<0.001	0.316	<0.001	-0.205	0.018
Endometrioma-Dermoid Cyst	-0.427	0.004	0.185	0.234	-0.162	0.298

AMH, Anti-Müllerian Hormone; FSH, Follicle-Stimulating Hormone; AFC, Antral Follicle Count; R, Correlation coefficient; P, P-value for significance testing; Cyst_Size, Size of the ovarian cyst; Endometrioma-Cyst_Size, The size of endometrioma cysts; Endometrioma-Simple Cyst, Comparison between endometrioma and simple cyst sizes; Endometrioma-Dermoid Cyst, Comparison between endometrioma and dermoid cyst sizes; The symbol “<“ denotes “less than.”;P-values listed as “<0.001” indicate that the results are statistically significant with a p-value less than 0.001.

### Regression analysis of cyst size and ovarian reserve markers

Multiple linear regression analysis (**Table 3**), adjusted for age and BMI, confirmed that increasing cyst size was independently associated with reduced AMH (β = –0.134, p < 0.001) and AFC (β = –0.346, p = 0.002), and elevated FSH levels (β = 0.455, p < 0.001). These effects were strongest in patients with endometriomas.

**Table 3:** Impact of ovarian cyst size on ovarian reserve indicators: regression analysis.

Variable	Coefficient (Coef.)	Standard error (Std.Err.)	t-value	P-value	95% Confidence interval (Lower)	95% Confidence interval (Upper)
AMH						
Constant	2.2594	0.1880	12.019	<0.0001	1.8891	2.6298
Cyst Size (cm)	-0.1337	0.0300	-4.451	<0.0001	-0.1929	-0.0745
AFC						
Constant	7.1381	0.5931	12.035	<0.0001	5.9697	8.3065
Cyst Size (cm)	-0.3452	0.0948	-3.642	0.0003	-0.5318	-0.1585
FSH						
Constant	12.0373	0.7069	17.029	<0.0001	10.6447	13.4298
Cyst Size (cm)	0.4546	0.1129	4.025	<0.0001	0.2321	0.6771

AMH, Anti-Müllerian Hormone; AFC, Antral Follicle Count; FSH, Follicle-Stimulating Hormone; Std.Err., Standard Error; Coef., Coefficient; CI, Confidence Interval.

## Discussion

This study provides comprehensive evidence of the significant impact of ovarian cyst type and size on ovarian reserve, particularly emphasizing the negative effects of endometriomas and larger cysts on key reproductive markers. Our findings indicate that both the type and size of ovarian cysts are critical determinants of ovarian function, with clear implications for fertility management.

### Impact of cyst type on ovarian reserve

Our data demonstrate that patients with endometriomas had significantly lower AMH levels and higher FSH levels compared to those with simple and dermoid cysts, as well as controls. This aligns with the understanding that endometriomas, associated with endometriosis, are particularly detrimental to ovarian reserve (14). The chronic inflammatory environment and fibrosis linked to endometriomas may cause more damage to the ovarian cortex, leading to a reduction in the functional ovarian reserve (15). In contrast, simple and dermoid cysts, while still impacting ovarian function, showed less severe effects on AMH and FSH levels. These findings suggest that endometriomas should be regarded as a more aggressive form of ovarian pathology with respect to reproductive health, and warrant earlier intervention or more careful monitoring, particularly in women who desire fertility preservation.

### Cyst size as a predictor of ovarian reserve decline

A key finding of this study is the strong inverse correlation between cyst size and AMH and AFC levels, coupled with a positive correlation between cyst size and FSH levels, regardless of cyst type. This suggests that larger cysts, irrespective of their classification, exert a more pronounced negative effect on ovarian function. The decline in AMH and AFC with increasing cyst size may be attributable to the mechanical disruption caused by the cyst or a more severe inflammatory response in cases of large endometriomas (16). These results highlight the importance of considering cyst size in clinical decision-making, especially when assessing the risk of diminished ovarian reserve. Moreover, our regression analysis, which controlled for age and BMI, confirmed that larger cysts are independently associated with lower AMH and AFC levels and elevated FSH levels, reinforcing the significance of cyst size as a predictor of ovarian reserve decline.

### Clinical implications for fertility management

The clinical implications of these findings are significant, particularly in the context of fertility preservation and treatment planning. Women with larger cysts, especially endometriomas, should be counseled on the potential risk of reduced ovarian reserve and may benefit from earlier fertility preservation strategies, such as oocyte or embryo cryopreservation. Additionally, the management of these cysts should be individualized based on their type and size. For example, surgical intervention may be considered in cases where cyst size is large or ovarian reserve is already compromised. However, the potential risks of surgery, such as further reduction in ovarian reserve due to the removal of healthy ovarian tissue, must be weighed carefully, particularly in women with a low baseline ovarian reserve.

For women with smaller cysts or benign cyst types such as simple or dermoid cysts, a more conservative approach may be appropriate. These patients, while still at risk of reduced ovarian function, may not require immediate surgical intervention. Instead, regular monitoring of ovarian reserve markers and cyst size should be implemented to guide clinical decisions. This approach minimizes the risk of overtreatment while ensuring that ovarian function is preserved as much as possible.

Furthermore, when managing fertility in women with endometriosis-related ovarian cysts, particularly endometriomas, a multidimensional approach is essential. As highlighted by Colombi et al. (2024), multiple factors—including the presence of pain symptoms, ovarian reserve status, prior surgeries, lesion bilaterality, age, and the urgency to conceive—must be jointly considered when choosing between surgical and assisted reproductive strategies (17). Surgical excision may benefit symptomatic patients with good ovarian reserve, while IVF is often preferred in cases of bilateral cysts, low AMH, or previous ovarian surgery due to the risk of further compromising ovarian function. The balance between spontaneous conception and IVF success, as well as postoperative risks such as reduced AMH levels, makes individualized and shared decision-making critical. Therefore, fertility-preserving strategies should be tailored based on both clinical and reproductive profiles to optimize outcomes in this complex patient population.

Additionally, surgical technique plays a critical role in preserving ovarian reserve following endometrioma excision. A recent meta-analysis by Wang et al. (2025) demonstrated that suture hemostasis causes significantly less reduction in AMH levels compared to bipolar electrocoagulation at 1, 3, 6, and 12 months postoperatively, with statistically significant differences favoring suturing at all time points (18). Although AMH levels partially recover over time in both groups, electrocoagulation results in more sustained impairment of ovarian reserve. These findings suggest that suturing may be a more conservative and fertility-preserving hemostatic approach, particularly in reproductive-age women. This supports the need for individualized surgical planning and reinforces the principle of minimizing thermal damage during ovarian surgery. The importance of meticulous hemostatic technique in gynecological procedures has long been emphasized, including early guidance from surgical nursing practice standards (19).

In addition to surgical considerations, anesthetic and analgesic techniques used during gynecologic procedures may influence the endocrine stress response and postoperative hormonal milieu. Recent studies have explored the role of regional anesthesia, such as transversus abdominis plane (TAP) blocks, in reducing perioperative stress and modulating endocrine markers. Romi et al. (2024) demonstrated that the use of dexmedetomidine as an adjuvant in TAP blocks significantly reduced postoperative serum cortisol levels, suggesting potential benefits for hormonal recovery following abdominal hysterectomy (20). Although these interventions do not directly alter ovarian reserve, they may contribute to an improved endocrine profile in the perioperative period. Furthermore, Riemma et al. (2021) reported comparable efficacy of TAP blocks and wound infiltration for post-cesarean analgesia, reinforcing the relevance of such regional techniques in gynecologic surgical care (21).

### Limitations and future directions

While this study offers important insights into the relationship between ovarian cysts and ovarian reserve, it is not without limitations. The retrospective design may introduce selection bias, and the study’s reliance on medical records may result in incomplete data for some patients. Additionally, this study was conducted in a single center, which may limit the generalizability of the findings to broader populations. One notable limitation of this study is that most ovarian cysts were diagnosed based on ultrasound findings without routine histopathological confirmation. Although we employed standardized sonographic criteria for classification, the absence of surgical pathology in the majority of cases may introduce a degree of diagnostic uncertainty. Future prospective, multicenter studies with larger and more diverse cohorts are needed to validate these findings and explore the long-term effects of ovarian cysts on fertility outcomes. Further research should also investigate the underlying biological mechanisms by which different cyst types and sizes influence ovarian reserve, which could help refine treatment strategies.

## Conclusion

In conclusion, this study underscores the significant impact of both cyst type and size on ovarian reserve markers, with larger cysts and endometriomas posing the greatest risk to ovarian function. These findings emphasize the need for a personalized approach to the management of ovarian cysts, particularly in women who are planning for pregnancy or considering fertility preservation. Careful assessment of cyst characteristics, along with regular monitoring of ovarian reserve, is essential for optimizing reproductive outcomes and ensuring the best possible fertility management for affected patients.

## Data Availability

The original contributions presented in the study are included in the article/**Supplementary Material**. Further inquiries can be directed to the corresponding author.
