# Supplementary material for: The influence of ovarian cyst type and size on ovarian reserve markers: implications for fertility counseling and preservation strategy
Source: Front Endocrinol (Lausanne). 2025 Jun 6;16:1517789. doi: 10.3389/fendo.2025.1517789 (PMC12178891; doi:10.3389/fendo.2025.1517789)
Supplement: Supplementary file 1 [file Table1.docx]

# STROBE Checklist – Filled

| Item No | Recommendation | Addressed in Manuscript (Yes/No/N/A) | Section/Page Reference |
| --- | --- | --- | --- |
| 1 | (a) Indicate the study’s design with a commonly used term in the title or the abstract | Yes | Title and Abstract |
| 1 | (b) Provide in the abstract an informative and balanced summary of what was done and what was found | Yes | Title and Abstract |
| 2 | Explain the scientific background and rationale for the investigation being reported | Yes | Introduction – Background |
| 3 | State specific objectives, including any prespecified hypotheses | Yes | Introduction – Objectives |
| 4 | Present key elements of study design early in the paper | Yes | Methods – Study Design |
| 5 | Describe the setting, locations, and relevant dates, including periods of recruitment, exposure, follow-up, and data collection | Yes | Methods – Setting |
| 6 | (a) Cohort study—Give the eligibility criteria, and the sources and methods of selection of participants. Describe methods of follow-up | Yes | Methods – Participants |
| 6 | (b) Cohort study—For matched studies, give matching criteria and number of exposed and unexposed | Yes | Methods – Age Matching (Participants) |
| 7 | Clearly define all outcomes, exposures, predictors, potential confounders, and effect modifiers. Give diagnostic criteria, if applicable | N/A | Not applicable |
| 8 | For each variable of interest, give sources of data and details of methods of assessment (measurement). Describe comparability of assessment methods if there is more than one group | Yes | Methods – Ovarian Reserve Assessment |
| 9 | Describe any efforts to address potential sources of bias | Yes | Discussion – Limitations |
| 10 | Explain how the study size was arrived at | Yes | Methods – Study Size |
| 11 | Explain how quantitative variables were handled in the analyses. If applicable, describe which groupings were chosen and why | Yes | Methods – Variables/Outcomes |
| 12 | (a) Describe all statistical methods, including those used to control for confounding | Yes | Methods – Statistical Methods |
| 12 | (b) Describe any methods used to examine subgroups and interactions | N/A | Not applicable |
| 12 | (c) Explain how missing data were addressed | Yes | Methods – Statistical Methods |
| 12 | (d) Cohort study—If applicable, explain how loss to follow-up was addressed | N/A | Retrospective Study – No follow-up |
| 12 | (e) Describe any sensitivity analyses | No | Not performed |
| 13 | (a) Report numbers of individuals at each stage of study | Yes | Results – Participants |
| 13 | (b) Give reasons for non-participation at each stage | No | Not addressed explicitly |
| 13 | (c) Consider use of a flow diagram | No | Flowchart not included |
| 14 | (a) Give characteristics of study participants and information on exposures and potential confounders | Yes | Results – Table 1 |
| 14 | (b) Indicate number of participants with missing data for each variable of interest | Yes | Methods – Statistical Methods |
| 14 | (c) Cohort study—Summarise follow-up time | N/A | Retrospective Study – No follow-up |
| 15 | Cohort study—Report numbers of outcome events or summary measures over time | Yes | Results – Ovarian Reserve Markers |
| 16 | (a) Give unadjusted and adjusted estimates and their precision | N/A | Not applicable |
| 16 | (b) Report category boundaries when continuous variables were categorized | Yes | Methods – Variables/Outcomes |
| 16 | (c) Translate estimates of relative risk into absolute risk if relevant | N/A | Not relevant to design |
| 17 | Report other analyses done—eg subgroup and sensitivity analyses | No | Not performed |
| 18 | Summarise key results with reference to study objectives | Yes | Introduction – Objectives |
| 19 | Discuss limitations of the study, including potential bias or imprecision | Yes | Discussion – Limitations |
| 20 | Give an overall interpretation of results considering limitations and other relevant evidence | Yes | Discussion – Limitations |
| 21 | Discuss the generalisability (external validity) of the study results | Yes | Discussion – Generalisability |
| 22 | Give the source of funding and the role of the funders | Yes | Funding Statement |
